# Surgical Procedures for External Auditory Canal Carcinoma and the Preservation of Postoperative Hearing

**DOI:** 10.1155/2012/841372

**Published:** 2012-12-01

**Authors:** Hiroshi Hoshikawa, Takenori Miyashita, Nozomu Mori

**Affiliations:** Head and Neck Surgery, Department of Otolaryngology, Faculty of Medicine, Kagawa University, 1750-1 Ikenobe, Miki-cho, Kita-gun, Kagawa 761-0793, Japan

## Abstract

Carcinoma of the external auditory canal (EAC) is an unusual head and neck malignancy. The pathophysiology of these tumors is different from other skin lesions because of their anatomical and functional characteristics. Early-stage carcinoma of the EAC can be generally cured by surgical treatment, and reconstruction of the EAC with a tympanoplasty can help to retain hearing, thus improving the patients' quality of life. In this study, we present two cases of early-stage carcinoma of the EAC treated by canal reconstruction using skin grafts after lateral temporal bone resection. A rolled-up skin graft with a temporal muscle flap was useful for keeping the form and maintaining the postoperative hearing. An adequate size of the skin graft and blood supply to the graft bed are important for achieving a successful operation.

## 1. Introduction

The majority of skin cancers of the head and neck are nonmelanoma skin cancers (NMSCs). Basal cell carcinoma and squamous cell carcinoma (SCC) are the most common types of NMSC. Carcinoma of the external auditory canal (EAC) is a rare form of malignancy encountered in otologic or head and neck surgical practices. The characteristic of these tumors are different from those of other skin lesions because of their pathogenesis and location. Although advanced carcinomas of the temporal bone, including the EAC, retain a poor prognosis, a good survival rate in earlier-stage patients was suggested to be possible in 100% of cases [1–3]. Therefore, the posttherapeutic functional and cosmetic preservation are of importance. In this study, we present two cases of early-stage EAC carcinoma and describe the surgical management and postoperative hearing and complications. 

## 2. Case Reports

### 2.1. Case 1

**Figure 1:**
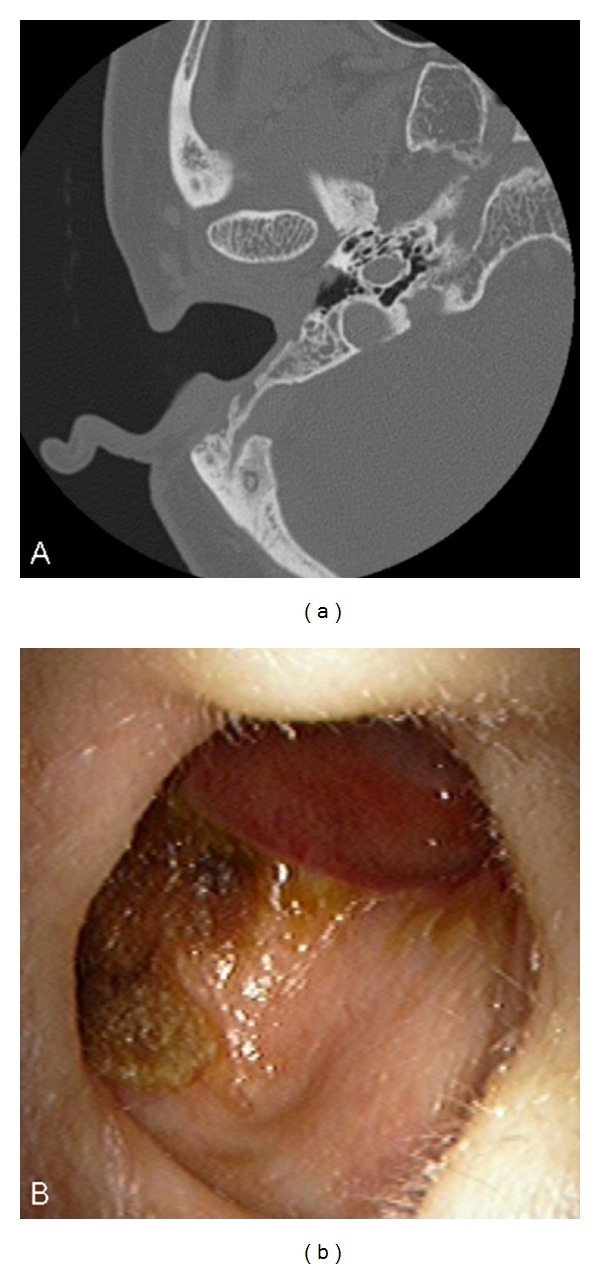
Six months after surgery in Case 1. (a) An axial CT image showed that the EAC had formed an open cavity with the mastoid bowl. (b) Although no stenosis or bone exposure was observed, a wet mucosal membrane and the crust were shown in the opened mastoid cavity.

A 59-year-old female presented with adenoid cystic carcinoma in the posterior-upper region of the EAC (T2N0M0). Magnetic resonance imaging (MRI) showed the tumor in the EAC, and no extension beyond the organ of origin was observed. Computed tomography (CT) showed evidence of bone destruction. No apparent lymph node swelling or distant metastasis was observed. Lateral temporal bone resection with preservation of the stapes was performed. A full thickness skin graft was taken from the posterior part of the auricle. The anterior and inferior parts of the EAC were covered by the skin graft, and a superior extremity of the skin graft was sutured to the inlet of the EAC. A piece of auricle cartilage was placed on the stapes head for conductive reconstruction, and the cartilage and mastoid cavity were sheeted with the fascia. The postoperative course was uneventful, and the graft skin survived completely. However, an opened mastoid cavity was formed, and a mucosal membrane was regenerated onto the surface of the cavity (Figure 1). Consequently, conservative treatment was needed in first two years. Pure-tone audiometry revealed deterioration of the postoperative hearing in the low frequency range. At the six-year checkup after surgery, no sign of a recurrent tumor was observed. 

### 2.2. Case 2

**Figure 2:**
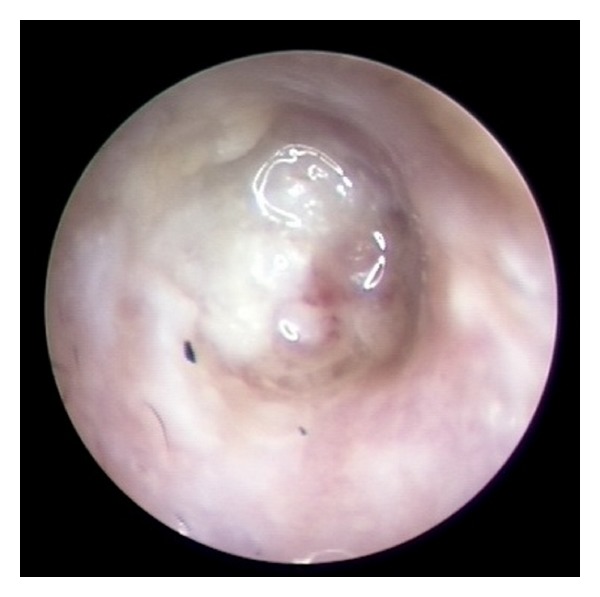
The first examination of the left ear in Case 2. Otorrhea and squamous debris with irregularity and granulation of the posterior ear canal down to the tympanic membrane was seen.

**Figure 3:**
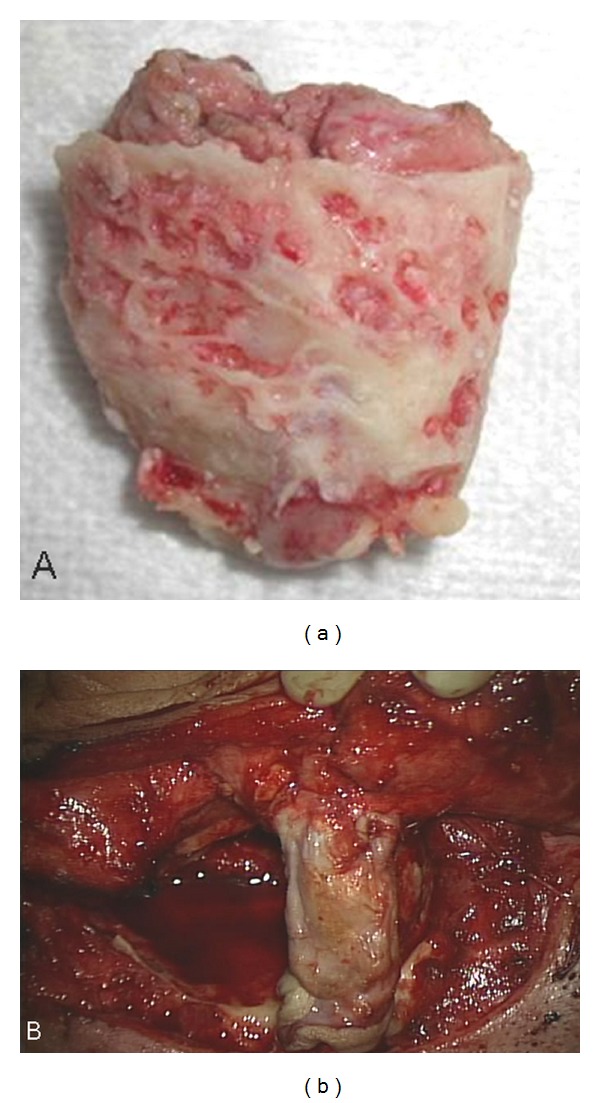
(a) Removal of the EAC with tumor. (b) The skin graft was rolled up and sutured with the inlet of the EAC and placed on the fascia as a substitute material for the tympanic membrane.

**Figure 4:**
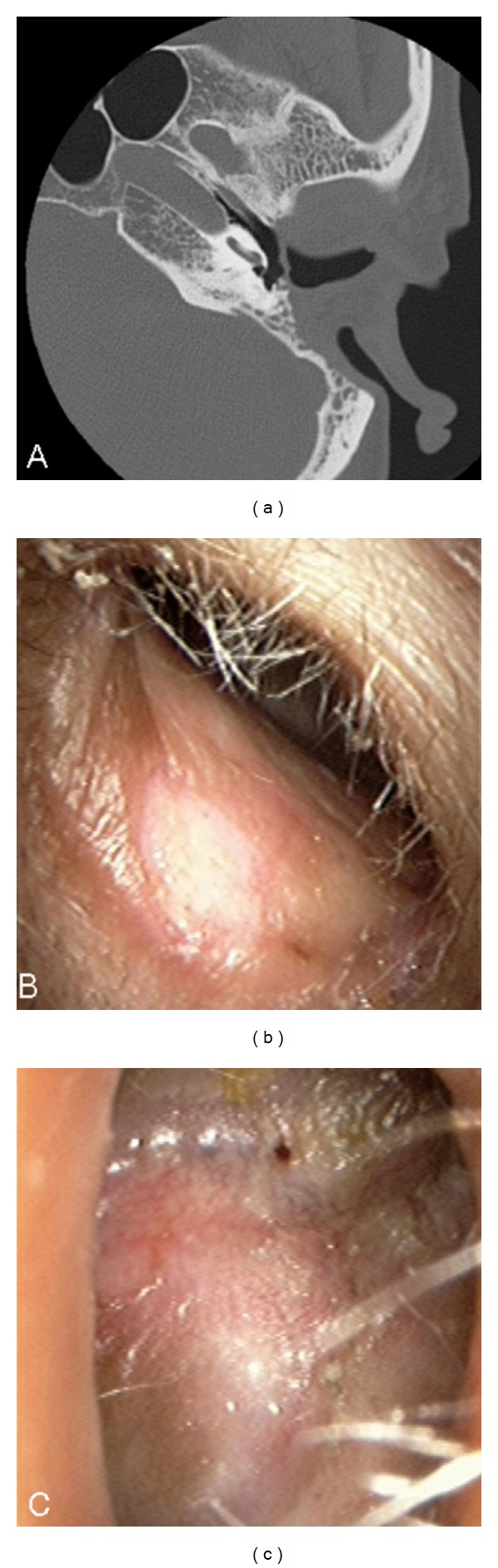
One-year postoperative findings in Case 2. (a) An axial CT image showed that the reconstructed EAC was kept in good shape, and no lateralization of the tympanic membrane was observed. (b and c) No stenosis or infection of the reconstructed external auditory canal was observed.

**Figure 5:**
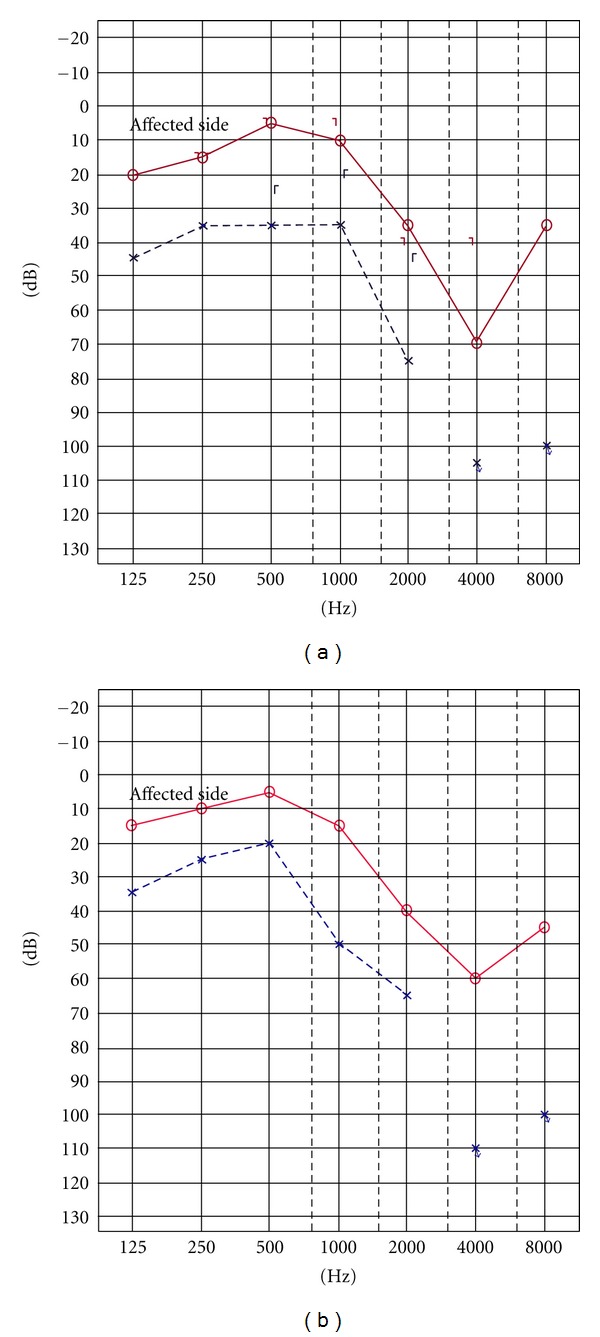
(a) Preoperative and (b) postoperative pure-tone audiogram. No hearing disturbance was observed after the surgery.

A 75-year-old male presented with SCC in the back of the EAC (Figure 2). CT imaging demonstrated no evidence of bony erosion and no extension beyond the middle ear or surrounding tissue (T1N0M0). Lateral temporal bone resection with preservation of the stapes was performed. Conductive reconstruction was performed using the auricular cartilage. A split thickness skin graft was rolled up as a tube using a gelatin sponge (Gelform) as a guide, and was inset into the defect. The superior extremity of the skin graft was sutured to the inlet of the EAC, and the inferior extremity of the skin graft was also sutured with fascia as a substitute material for the tympanic membrane (Figure 3). The anterior and inferior parts of the rolled-up skin graft were attached to surrounding soft tissue by fibrin glue, and the superior and posterior parts of the graft were covered by a pedicle muscle flap. The postoperative course was uneventful, and the graft skin survived completely. Complete patency of the reconstructed EAC was accomplished (Figure 4), and no hearing disturbance was observed after the surgery (Figure 5). No stenosis or recurrent tumor had been observed as of four years after surgery.

## 3. Discussion

Although several types of tumors have been reported in the external auditory canal, such as basal cell carcinoma [4], ceruminous carcinoma, undifferentiated carcinoma, malignant melanoma [5] and, mucoepidermoid carcinoma [6], SCC and adenoid cystic carcinomas are the most common types of EAC cancers [1]. The early identification of these tumors is essential to limit tumor extension. A relationship between the extent of disease and patient survival has been clearly demonstrated [7]. On the other hand, a good survival in earlier-stage cancer was suggested, with rates as high as 100% [1–3]. 

Not only the complete resection, but also reconstruction of the auditory canal is necessary to maintain the quality of life for the patients. Several methods using free flaps have been reported for the reconstruction of the EAC after tumor resection [8–10]. The advantages of these methods are that they can prevent stenosis, delayed wound healing, chronic infection, and bone exposure. However, skin grafting is most commonly performed because of its technical simplicity and shorter operation time. In the present study, we presented two types of reconstruction with auditory canal and mastoid cavity using a skin graft technique. In Case 1, the anteroinferior parts of the ear canal were reconstructed by skin, and the supraposterior parts of the EAC spread to the mastoid cavity. Postoperative care was needed for first two years because of secretions from the regenerated mucosa of the mastoid cavity. Deterioration of low frequency hearing was also noted. In Case 2, the split thickness skin graft was rolled up as a tube and inserted into the defect. In this case, an adequate length of skin graft was prepared to prevent lateralization of the tympanic membrane, and one-third to one-half of the posterior temporal muscle flap was rotated as a graft bed to decrease the size of the open mastoid cavity. As a result, complete patency of the reconstructed EAC was accomplished, and no hearing disturbance was observed after the surgery.

In conclusion, rolled-up skin grafting is therefore considered to be useful for reconstruction of the EAC and preservation of postoperative hearing. An adequate length of skin graft and blood supply with a graft bed are important for a successful operation.
